# Supplementary material for: Notoginsenoside R1 Attenuates Cisplatin-Induced Ototoxicity by Inducing Heme Oxygenase-1 Expression and Suppressing Oxidative Stress
Source: Int J Mol Sci. 2024 Oct 24;25(21):11444. doi: 10.3390/ijms252111444 (PMC11546915; doi:10.3390/ijms252111444)
Supplement: Supplementary file 1 [file ijms-25-11444-s001.zip › ijms-3245668-supplementary.pdf]

Supplementary Figure S1:

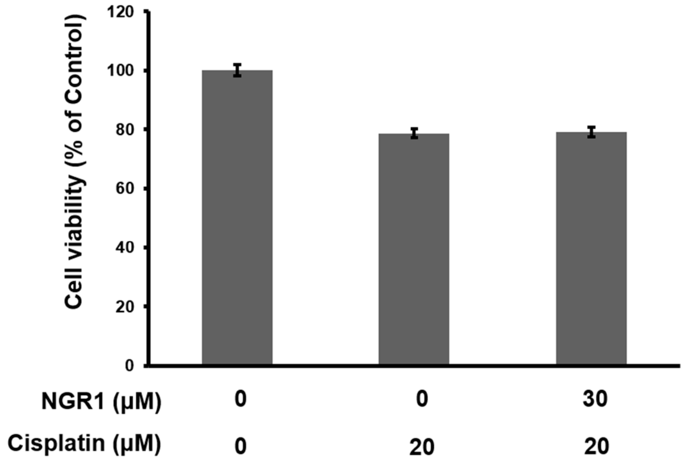

Figure S1. Viability of HEI-OC1 cells treated with 30 μM NGR1 and cisplatin. The cells were treated with 20 μM cisplatin alone for 24 h or pretreated with NGR1 for 24 h, followed by 24 h of cotreatment with NGR1 and cisplatin. n = 12 for each group.
